# Comparison of region-of-interest delineation methods for diffusion tensor imaging in patients with cervical spondylotic radiculopathy

**DOI:** 10.1186/s12891-022-05639-5

**Published:** 2022-07-15

**Authors:** Penghuan Wu, Chengyan Huang, Benchao Shi, Anmin Jin

**Affiliations:** 1 Shaoguan First People’s Hospital, Affiliated Shaoguan First People’s Hospital, Southern Medical University, Guangdong, China; 2 Department of Radiology, Zhujiang Hospital, Southern Medical University, Guangzhou, China; 3 Department of Spinal Surgery, Orthopedics Center, Zhujiang Hospital, Southern Medical University, Guangzhou, China

**Keywords:** Diffusion tensor imaging, Cervical radiculopathy, Imaging parameters

## Abstract

**Background:**

Diffusion tensor imaging is a promising technique for determining the responsible lesion of cervical radiculopathy, but the selection and delineation of the region of interest (ROI) affect the results. This study explored the impact of different ROI sketching methods on the repeatability and consistency of DTI measurement values in patients with cervical spondylotic radiculopathy (CSR).

**Methods:**

This retrospective study included CSR patients who underwent DTI imaging. The images were analyzed independently by two radiologists. Four delineation methods were used: freehand method, maximum roundness, quadrilateral method, and multi-point averaging method. They re-examined the images 6 weeks later. The intra-class correlation coefficient (ICC) was used to investigate the consistency between the two measurements and the reproducibility between two radiologists.

**Results:**

Forty-two CSR patients were included in this study. The distribution of the compressed nerve roots was five C4, eight C5, sixteen C6, eleven C7, and two C8. No differences were found among the four methods in fractional anisotropy (FA) or apparent diffusion coefficient (ADC), irrespective of radiologists (all P>0.05). Similar results were observed between the first and second measurements (all P>0.05), but some significant differences were observed for radiologist 2 for the four-small rounds method (P=0.033). The freehand and single largest circle methods were the two methods with the highest ICC between the two measurements and the two radiologists (all ICC >0.90).

**Conclusion:**

The freehand and single largest circle methods were the most consistent methods for delineating DTI ROI in patients with CSR.

## Background

Cervical radicular pain is an impingement of a cervical spinal nerve and/or nerve root typically characterized by unilateral shooting electric pain in the upper limb, and, if radiculopathy, sensory, motor, and/or reflex deficits; nonspecific symptoms include neck pain [1–3]. When neurological symptoms appear or if conservative treatment is ineffective, neurodecompression surgery has to be performed in time [4, 5].

Patients with cervical nerve root radiation pain often encounter problems during management. First, preoperative imaging findings are inconsistent with the symptoms and signs [6]. Second, because of multi-segment cervical disc herniation or spinal canal stenosis, the exact responsible segment cannot be determined [7]. Third, even though electrophysiological tests are widely used, it is difficult to distinguish the responsible lesion from other cervical peripheral nerve injury diseases (such as ulnar neuritis, cubital tunnel syndrome, carpal tunnel syndrome, etc.) and shoulder joint-related diseases. With the continuous development of minimally invasive spine surgery, accurately identifying the responsible lesion, accurately judging the responsible segment and location, and achieving accurate diagnosis and treatment are challenges for spinal surgeons.

At present, clinical routine computed tomography (CT) and magnetic resonance imaging (MRI) examinations can reveal the location of cervical disc herniation and fibrous bone canal stenosis, thereby indirectly determining whether the nerve is compressed and injured, but they cannot provide direct evidence of nerve root injury [8–10]. In addition, imaging can be assisted by neuroelectrophysiological examination and nerve root block to better judge the responsible segment. Nevertheless, these examinations are invasive, their specificity and sensitivity are open to question [11], and their clinical application is limited due to the risk of complications [12]. Therefore, there is an urgent need for non-invasive, accurate, and operable examination methods that could qualitatively and quantitatively reflect the degree and location of nerve root injury.

Diffusion tensor imaging (DTI) is a diffusion-weighted imaging (DWI) sequence based on multiple b-values and can quantitatively analyze the diffusion of water molecules in living tissues [13]. The main parameters include fractional anisotropy (FA) and apparent diffusion coefficient (ADC). FA is represented by a scalar value between 0 and 1 that describes the degree of diffusion. An FA value of 0 means that the diffusion is isotropic, i.e., unrestricted (or equally restricted) in all directions; an FA value of 1 means that diffusion occurs only along one axis and is fully restricted along all other directions [14–16]. FA is often used in diffusion imaging to reflect fiber density, axonal diameter, and white matter myelination [14–16]. ADC considers that the diffusion of water molecules is complex in biological tissues and reflects several different mechanisms [14–16]. Therefore, since the movement of water molecules in the nerve tissue is along the nerve fibers with anisotropic dispersion, the DTI technique can be used in theory to evaluate the pathological changes of nerve roots [17] and quantify the changes [18]. DTI has been widely used to diagnose central nervous system diseases [19]. Chen et al. [20] reported that DTI could be used to assess the microstructural abnormalities in the cervical nerve roots in patients with disc herniation.

The study of the cervical nerve root system by the DTI technique is still in its initial stage. One of the most challenging aspects is the proper selection and delineation of the region of interest (ROI) [21, 22]. Indeed, the ROI size considerably influences tumors’ ADC values [23, 24]. Therefore, this study aimed to explore the impact of different ROI sketching methods on the repeatability and consistency of DTI measurement values in patients with cervical spondylotic radiculopathy. The results could help develop the appropriate method and lay an objective and solid theoretical foundation for subsequent studies.

## Methods

### Study design and patients

This retrospective study included CSR patients who underwent imaging from May 2016 to May 2018 at the Department of Spine Surgery, Zhujiang Hospital of Southern Medical University. The diagnostic criteria of cervical spondylotic radiculopathy were typical root symptoms (arm numbness or pain), which were consistent with the area of a cervical spinal nerve, and the brachial plexus tension test or foraminal compression test was positive [2]. In all the patients, the location of symptoms (e.g., dermatomal pain or neurological deficit) matched the evaluated nerve root on the DTI images. This study was approved by the Institutional Review Board of Zhujiang Hospital of South Medical University (Guangzhou, China). The need for individual consent was waived by the committee because of the retrospective nature of the study.

The inclusion criteria were 1) diagnosis of cervical spondylotic radiculopathy, as above, 2) symptoms and signs of unilateral cervical nerve root compression, and 3) radiological clues of single-level, unilateral posterolateral protrusion into the intervertebral foramen of the intervertebral disc. The exclusion criteria were 1) a previous history of spinal trauma or surgery, 2) a history of neurological disease, 3) a history of chronic infection, or 4) a history of claustrophobia or any psychological problems.

### DTI

**Fig. 1:**
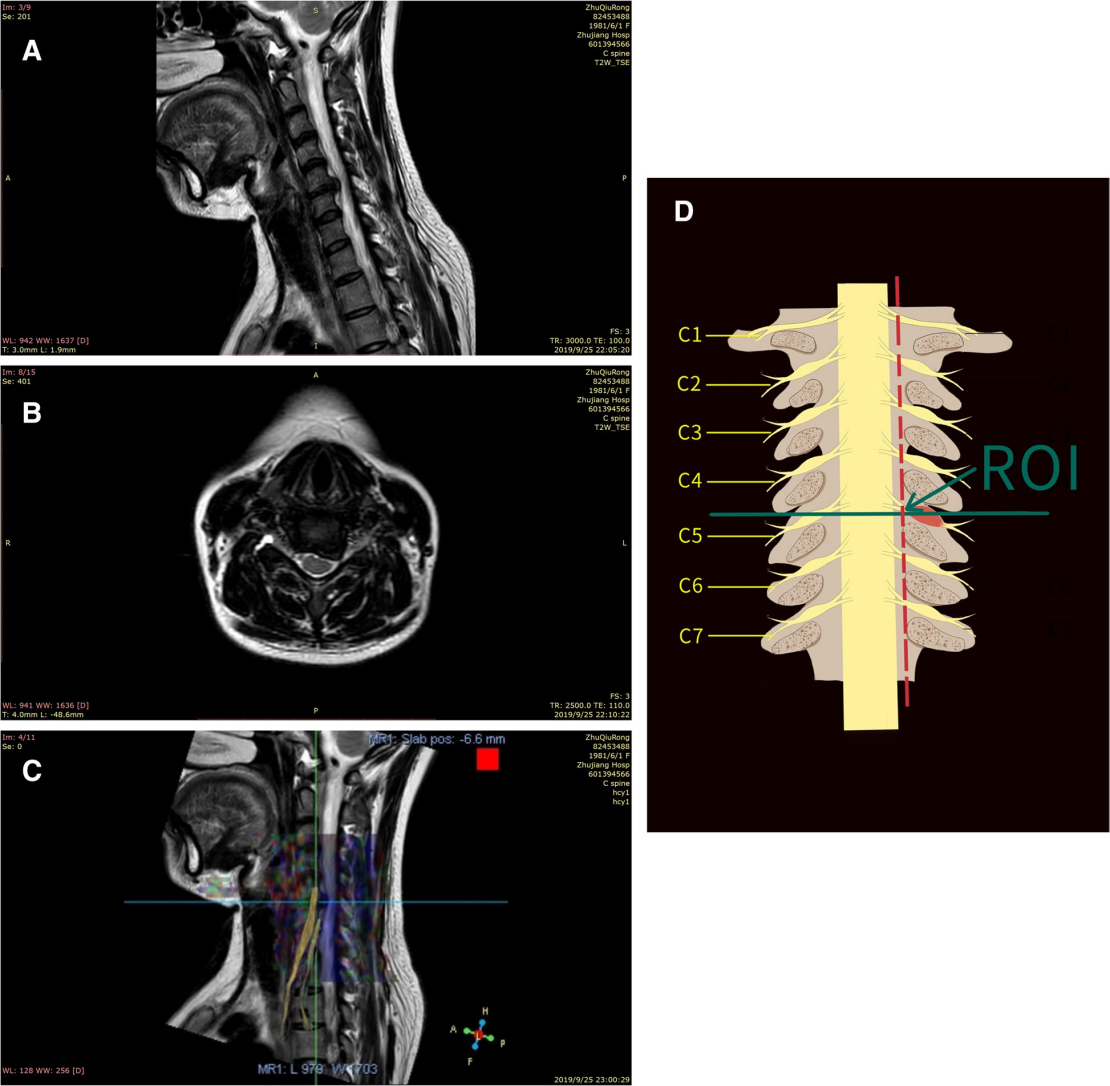
The patient was 45 years old, male, with left upper limb pain and numbness for 8 months. The cervical 5-6 disc was herniated and presented with numbness and pain in the left lateral forearm and left middle finger. **A** Cervical 5-6 disc herniation, sagittal plane. **B** Left intervertebral foramen stenosis, axial plane. **C** Diffusion-tensor imaging (DTI) positioned the injured nerve root at 5-6 cervical segments. **D** The measuring plane of regions of interest (ROI) was placed at the entrance to the intervertebral foramen, i.e., the intersection of the attachment of the medial edge of the affected superior pedicle to the inferior pedicle and the nerve root

All patients underwent DTI in the supine position using a 3-T scanner (Ingenia, Philips, The Netherlands) with a 6-channel head and neck coil. The coil was positioned at the center of the mandible, and the scanning interval was between C2 and T1. The DTI scan was at the axial position before the sagittal and coronal planes were constructed. Two radiologists (6 and 5.5 years of experience in spine MRI) supervised the examinations. The imaging parameters were b value, 0 and 800 s/mm^2^; directions, 32; TR/TE, 4500/67 ms; orientation, axial; slice thickness/gap, 2/1 mm; FOV, 224×224 mm^2^; actual voxel size, 2.5×2.9×2 mm^3^; total slices, 30; and scan time, 9 min 49 s. Axial T2-weighted anatomical images were also obtained using the T2 turbo-spin-echo (TSE) sequence: variable flip angle radiofrequency excitations; TR/TE, 2500/110 ms; FOV, 155×155 mm; and section thickness/gap, 15/1.2 mm. A typical case is shown in Fig. 1A-B.

### Image processing and analysis

The DTI images were transferred to the EWS4.1 workstation and measured by two radiologists (6 and 5.5 years of experience in spine MRI) independently using the Philips post-processing software (Fig. 1C). In order to determine the level of entrance to the intervertebral foramen, an anatomic fusion of the DTI and T2WI-weighted images was performed for the intersection of the attachment of the medial edge of the affected superior pedicle to the inferior pedicle and the nerve root (Fig. 1D). The ROI was set on the B0 images.

**Fig. 2:**
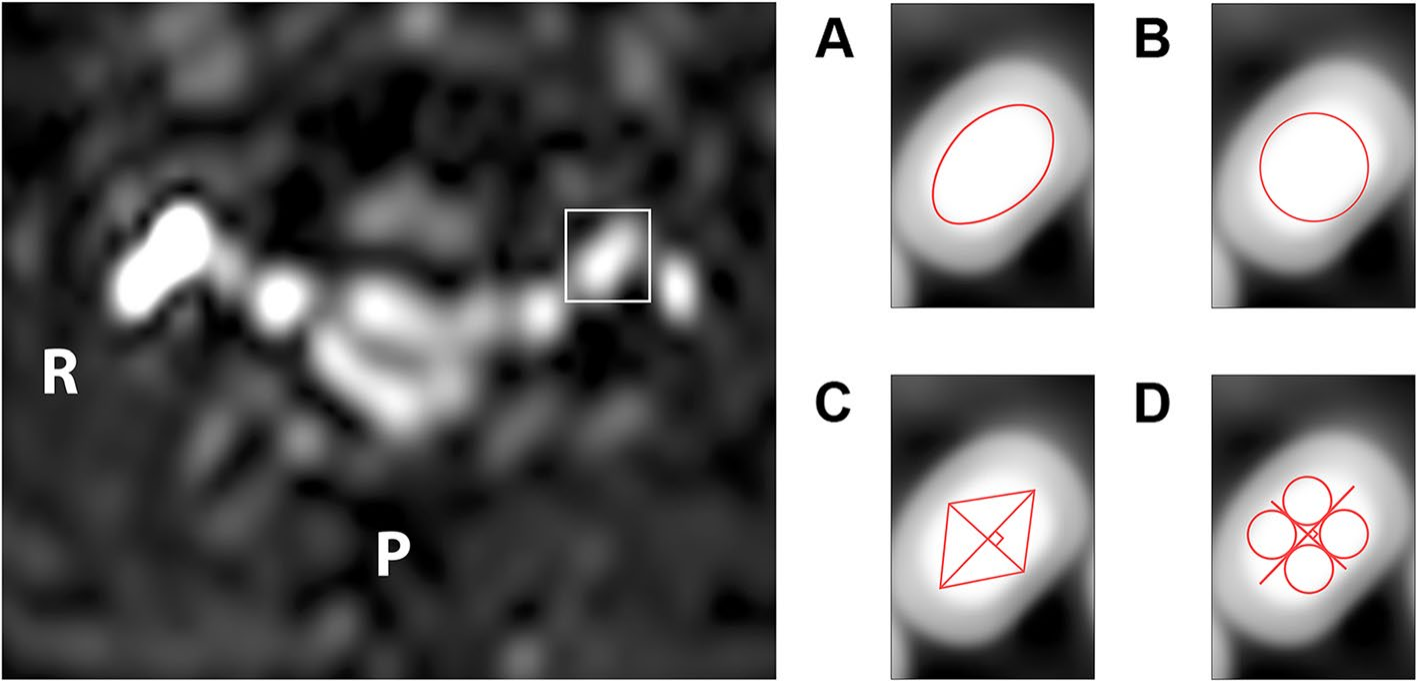
Four methods of region of interest (ROI) sketching. **A** The freehand method. **B** The maximum roundness method. **C** The quadrilateral method. **D** The multi-point averaging method

The FA and ADC values were measured as follows: B=800 image high signal area, fusing 3D-FFE image to assist localization, and sketched four ROIs at the entrance level to the intervertebral foramen. Four delineation methods were used: 1) freehand method (Fig. 2A): manually tracing the ROI along the nerve root contour to avoid cerebrospinal fluid interference [25, 26]; 2) maximum roundness (Fig. 2B): the round ROI was drawn as large as possible, tangential to the edge of the nerve root, covering the maximum nerve root area and not exceeding the edge [27]; 3) quadrilateral method (Fig. 2C): the longest axis of the nerve root section area was first drawn, followed by the vertical axis, and then the endpoints of the two axes were connected clockwise to form a quadrilateral; and 4) the multi-point averaging method (Fig. 2D): the above two axes above divide the nerve roots into four quadrants, the largest circle was drawn in each quadrant, the average value of the four circles was taken as the measured value of DTI [28, 29]. The corresponding cervical 4-8 nerve root reconstructions were generated. Six weeks later, the two radiologists repeated the measurements.

### Statistical analysis

All analyses were performed using SPSS 25.0 (IBM, Armonk, NY, USA). The continuous data were presented as means ± standard deviations (SD). The categorical data were presented as n (%). For the repeated measurements between the two radiologists, the comparisons were made using the paired t-test. For the overall differences among the four methods, one-way repeated measurement ANOVA was used with the LSD post hoc test. The intra-class correlation coefficient (ICC) was used to investigate the consistency between the two measurements and the reproducibility between the two radiologists. The model of ICC was set as two-way random, which considers both rater and participant error. The ICC type was set as an absolute agreement. The R software (version 3.5.2) and ‘BlandAltmanLeh’ package were used to obtain the Bland-Altman plots. A P-value <0.05 was considered statistically significant (two-tailed).

## Results

### Baseline characteristics of the patients

Fifty-six patients were enrolled, and 42 patients were finally included. The exclusion reasons were: the nerve root volume was too small, and the DTI images were unclear (*n*=6); poor quality of DTI and the T2 images (*n*=5); imaging could not be completed (*n*=3). The characteristics of the patients are shown in Table 1. The mean age was 51.8±6.1 years (range: 37 to 65). The sex ratio was 1:0.83 (male/female=23/19). The distribution of the compressed nerve roots was five C4, eight C5, 16 C6, 11 C7, and two C8.

**Table 1:** Clinical characteristics of the patients

Parameters	Mean ± SD / n (%)
Age, years	51.8 ± 6.1
Sex	
Male	23 (54.8%)
Female	19 (45.2%)
Compressed nerve root	
C4	5 (11.9%)
C5	8 (19.1%)
C6	16 (38.1%)
C7	11 (26.2%)
C8	2 (4.8%)

*SD* standard deviation

### FA and ADC measurement results

The size of the ROIs was 34.7±12.5 mm^2^ using the freehand method, 34.2±11.3 mm^2^ using the maximum roundness method, 33.9±10.8 mm^2^ using the quadrilateral method, and 33.6±13.4 mm^2^ using the multi-point averaging method. Table 2 shows the results of all FA and ADC measurements. No significant differences were found among the four methods in FA or ADC, irrespective of radiologists (all *P*>0.05). Similar results were observed between the first and second measurements (all *P*>0.05), but some significant differences were observed for radiologist 2 for the four-small rounds method (*P*=0.033).

**Table 2:** FA and ADC results of the two technicians and two time points

	FA				ADC (10^-3^ mm^2^/s)			
	Pre	Post	P	ICC*	Pre	Post	P	ICC^a^
Radiologist 1								
Free hand	0.22±0.04	0.22±0.03	0.479	0.904 (0.829 to 0.947)	1.54±0.21	1.54±0.18	0.949	0.934 (0.880 to 0.964)
The single largest round	0.22±0.03	0.22±0.03	0.623	0.975 (0.955 to 0.987)	1.56±0.15	1.56±0.15	0.121	0.984 (0.971 to 0.991)
Single rectangle	0.21±0.03	0.21±0.03	0.916	0.892 (0.807 to 0.940)	1.56±0.16	1.57±0.15	0.572	0.920 (0.856 to 0.956)
Four-small rounds	0.22±0.03	0.21±0.03	0.213	0.793 (0.648 to 0.883)	1.55±0.15	1.56±0.14	0.369	0.796 (0.652 to 0.885)
P	0.955	0.942			0.941	0.847		
Radiologist 2								
Free hand	0.22±0.03	0.22±0.03	0.914	0.905 (0.829 to 0.948)	1.54±0.18	1.55±0.18	0.533	0.937 (0.886 to 0.966)
The single largest round	0.22±0.03	0.22±0.03	0.781	0.916 (0.849 to 0.954)	1.56±0.15	1.56±0.14	0.709	0.960 (0.927 to 0.978)
Single rectangle	0.21±0.03	0.22±0.03	0.215	0.671 (0.466 to 0.808)	1.57±0.16	1.55±0.16	0.133	0.866 (0.764 to 0.926)
Four-small rounds	0.21±0.03	0.21±0.02	0.726	0.779 (0.624 to 0.875)	1.56±0.16	1.53±0.15	0.033	0.755 (0.580 to 0.862)
P	0.867	0.930			0.882	0.737		

*FA* fractional anisotropy, *ADC* apparent diffusion coefficient, *ICC* intra-class correlation coefficient. ^a^ICC analysis for both FA and ADC of different measurements were all significant (*P*<0.001)

Within each radiologist, the ICC was used to investigate the consistency of the repeated measurements. As indicated in Table 2, all results were positively significant (all P<0.001), indicating high consistency. The freehand and single largest circle were the two methods with the highest ICC (all ICC >0.90).

### FA and ADC measurement consistency between two radiologists

**Fig 3:**
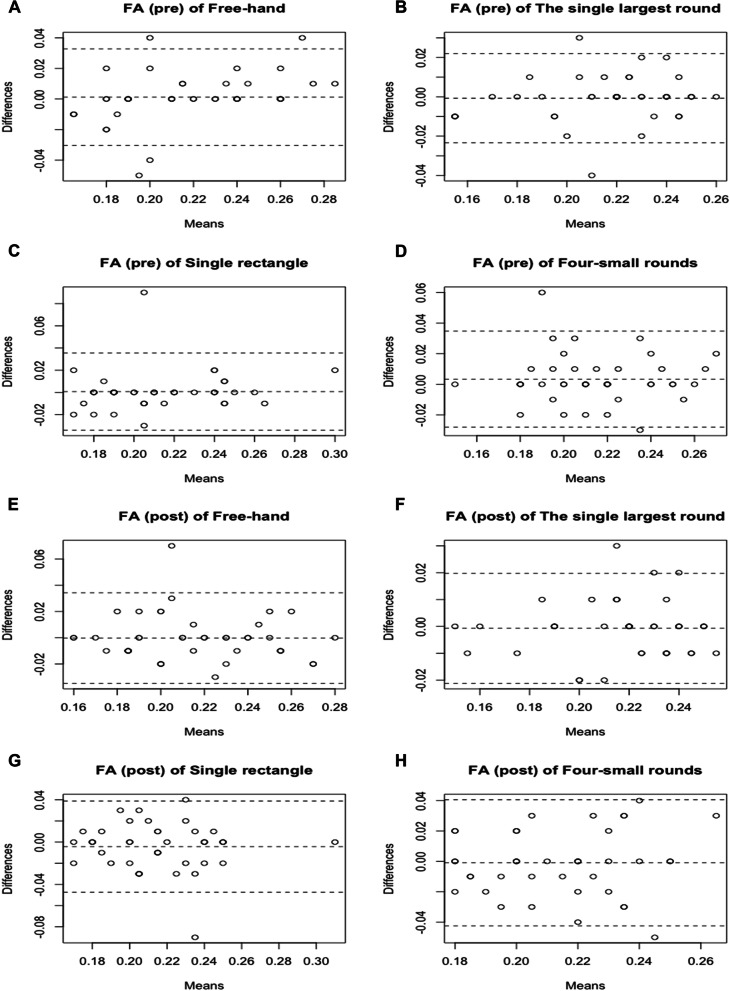
Bland-Altman plots of fractional anisotropy (FA) between the two technicians, including pre-measurements of freehand **A**, the single largest round **B**, single rectangle **(C)**, four-small rounds **(D)**; and post-measurements of freehand **(E)**, the single largest round **(F)**, single rectangle **(G)**, four-small rounds **(H)**

**Fig. 4:**
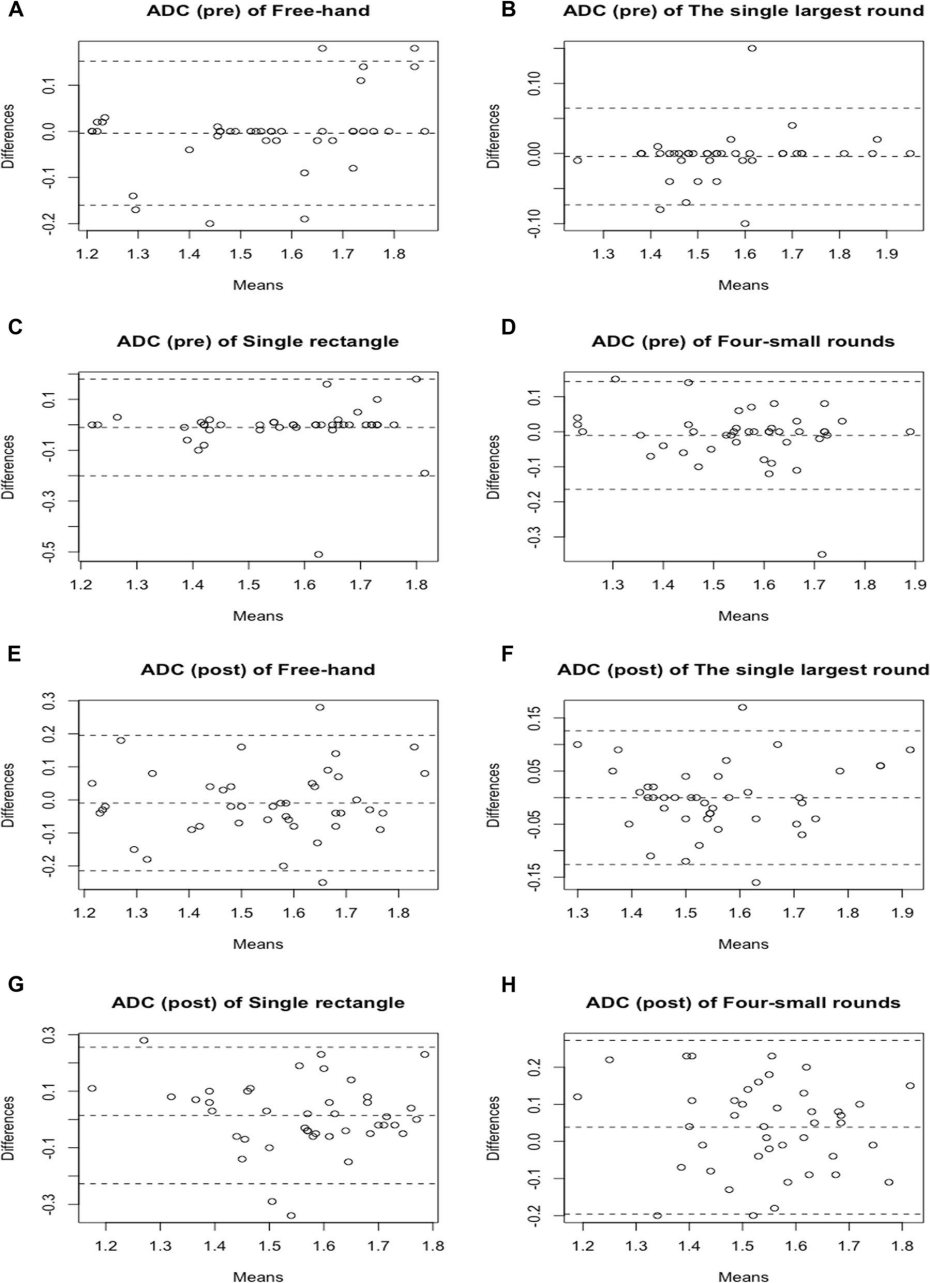
Bland-Altman plots of apparent diffusion coefficient (ADC) between the two technicians, including pre-measurements of freehand **(A)**, the single largest round **(B)**, single rectangle **(C)**, four-small rounds **(D)**; and post-measurements of freehand **(E)**, the single largest round **(F)**, single rectangle **(G)**, four-small rounds **(H)**

The ICC between the two radiologists was used to investigate the reproducibility of the measurements between investigators. As indicated in Table 3, all results were significant (all *P*<0.001), but the ICC for the freehand and the single largest round methods were higher than for the two other methods (all ICC >0.90). The Bland-Altman plots in Figs. 3 and 4 also indicated similar results to the numeric statistics. The freehand method had differences gathered around zero (mean of -0.010±0.105 to 0.001±0.016), fewer outliers (*n*=1-4), and well-distributed plots. The same was observed for the single largest round method (mean of -0.001±0.012 to -0.000±0.064; 2-3 outliers) compared with the other two methods.

**Table 3:** ICC results between radiologists 1 and 2

	FA		ADC	
Radiologists 1 and 2	ICC	P	ICC	P
Measurement 1				
Free hand	0.889 (0.804 to 0.939)	<0.001	0.918 (0.853 to 0.955)	<0.001
The single largest round	0.914 (0.846 to 0.953)	<0.001	0.973 (0.950 to 0.985)	<0.001
Single rectangle	0.839 (0.720 to 0.910)	<0.001	0.817 (0.686 to 0.897)	<0.001
Four-small rounds	0.832 (0.709 to 0.906)	<0.001	0.872 (0.775 to 0.929)	<0.001
Measurement 2				
Free hand	0.858 (0.751 to 0.921)	<0.001	0.833 (0.711 to 0.907)	<0.001
The single largest round	0.926 (0.866 to 0.959)	<0.001	0.904 (0.828 to 0.947)	<0.001
Single rectangle	0.734 (0.559 to 0.847)	<0.001	0.684 (0.484 to 0.817)	<0.001
Four-small rounds	0.642 (0.421 to 0.791)	<0.001	0.650 (0.432 to 0.796)	<0.001

*FA* fractional anisotropy, *ADC* apparent diffusion coefficient, *ICC* intra-class correlation coefficient

## Discussion

DTI is a promising technique for determining the responsible lesion of cervical radiculopathy [30], but the selection and delineation of the ROI influence the results [23, 24]. Currently, there are no published studies on ROI delineation methods in nerve root measurement. Wu et al. [28] and Wang et al. [29] used the multi-point averaging method for the evaluation of the spinal cord and the grading of cervical spondylotic myelopathy, and Zhu et al. [25] and Hakulinen et al. [26] used the freehand method for the evaluation of cervical spinal cord trauma and brain matter, respectively. These four studies did not examine specifically the nerve roots, and they did not compare different delineation methods. Inoue et al. [27] compared four different methods (freehand ROI, square ROI, round ROI, and five small, round ROIs) for evaluating endometrial carcinoma and proposed the maximum roundness method as being the most optimal one. Unlike normal nerve roots, diseased nerve roots are compressed in different directions, resulting in different degrees of edema inside the nerve root (nerve fibers), so the signals of the nerve root cross-section are heterogeneous. Different drawing methods cover different signal areas, leading to different DTI values. Therefore, it is of clinical significance to discuss the ROI sketching method for diseased nerve roots. The results should be important for improving the evaluation of the patients.

This study aimed to explore the impact of different ROI sketching methods on the repeatability and consistency of DTI measurement values in patients with cervical spondylotic radiculopathy. The results suggest that for the delineation of DTI ROI in patients with cervical spondylotic radiculopathy, the freehand and single largest circle methods were the most consistent.

In this study, we found that when measuring FA and ADC in patients with cervical spondylotic radiculopathy, intra- and interobserver variabilities were dependent upon the methods of ROI delineation. FA and ADC measurements obtained by the largest circle and the freehand methods were more reproducible than those obtained from the rectangle or four small round measurements. Ma et al. [31] found that the ROI size had a considerable influence on the ADC measurements of PDACs and suggested that the largest round ROI yielded the best intra- and interobserver reproducibility. At the same time, Jafari-Khouzani et al. [32] suggested that increasing the ROI size could reduce the FA and ADC values variance. It is consistent with our results. We think that a larger circle will yield high repeatability and consistency. Of course, the nerve root cross-section is mostly a circle. A round ROI covering the cross-sectional nerve area will include most pixels, leading to the highest homogeneity. Moreover, the maximum circular ROI is easier to operate in practice with less time and better controllability, which greatly reduces the measurement errors caused by the circle sketched beyond the actual boundary of the nerve root during the actual operation. Therefore, the ICCs are high. The ROIs area of the freehand method is greater than the maximum roundness method. Nevertheless, due to the time-consuming drawing process, the contour method requires the operator to continuously judge and identify the actual boundary of the nerve roots. Therefore, the result is greatly affected by the operator’s subjective factors.

The quadrilateral and four small circles ROI methods have high central variability and lower ICCs. It takes longer to include fewer pixels. Nogueira et al. [24] report that small ROIs show high ADC reproducibility in the DTI diagnosis of breast lesions. Inoue et al. [27] showed that the ROI shape has no marked influence on the ICC in endometrial carcinoma. These studies contradict our conclusions and may be due to the different nature of the subjects and lesions. Moreover, an important advantage of using the largest round ROI is that its placement is much less time-consuming than delimiting the whole slice. Lambregts et al. [21] and Ma et al. [31] showed that the ROI has a considerable influence on tumor DTI values. Sun et al. [33] showed that the ADC and FA values derived from outline ROIs are higher than those from round ROIs. Inconsistent with those previous studies, we found no significant differences in the FA values and ADC values among the four methods, which may be related to the small sample size. Moreover, FA and ADC values may be correlated with age, sex, and BMI. The cross-sectional areas of the nerve roots included in the four methods were similar. The obtained FA measurements of the four ROIs are lower than those reported in the previous literature [20, 30], which might be because the included patients had more severe root compression than the reported patients. In the present study, the mean FA values in the entrapped nerve roots were lower than they were in the intact nerve roots, indicating that diffusion in the tissue had become more isotropic because of edema, in which fluid is trapped in the tissue, creating an isotropic environment and a reduction in FA.

Of course, there are limitations to this study. First, to obtain the typical diseased nerve root, we adopted very strict case inclusion criteria, leading to a small sample size. Second, due to the limited sample size, we did not group the patients according to sex, age, body mass index, and other factors and did not study the possible influence of different factors on the results. In addition, due to the small sample size, the confounding effects of age, sex, deterioration, and other confounding variables could overestimate or underestimate the DTI parameters. Third, only patients with unilateral compression were included, which could decrease the generalizability of the results. Fourth, this study examined the ADC and FA values but not fiber tracing. Therefore, in future studies, we will combine multiple centers to screen and include typical cases and assess the influence of relevant factors on the results. The present study is the first of a series of studies with the ultimate goal of using DTI to directly diagnose cervical spondylotic radiculopathy. This study is the first step, aiming at the selection of the ROI method for delineating diseased nerve roots rather than healthy nerve roots. Future studies will include healthy volunteers.

## Conclusions

In conclusion, the results suggest that for the delineation of DTI ROI in patients with cervical spondylotic radiculopathy, the freehand and single largest circle methods were the most consistent methods. The results provide a reliable and objective basis for the use of DTI in the diagnosis and treatment of cervical spondylotic radiculopathy.

## Data Availability

The datasets used and/or analyzed during the current study are available from the corresponding author on reasonable request.

## Abbreviations

ROI: region of interest
CSR: cervical spondylotic radiculopathy
ICC: intra-class correlation coefficient
FA: fractional anisotropy
ADC: apparent diffusion coefficient
CT: computed tomography
MRI: magnetic resonance imaging
DTI: Diffusion tensor imaging
DWI: diffusion-weighted imaging
SD: standard deviations

## References

[1] Childress MA, Becker BA (2016). Nonoperative Management of Cervical Radiculopathy. Am Fam Physician.

[2] Bono CM, Ghiselli G, Gilbert TJ, Kreiner DS, Reitman C, Summers JT, Baisden JL, Easa J, Fernand R, Lamer T (2011). An evidence-based clinical guideline for the diagnosis and treatment of cervical radiculopathy from degenerative disorders. Spine J.

[3] Van Zundert J, Huntoon M, Patijn J, Lataster A, Mekhail N, van Kleef M (2010). Pain P: 4. Cervical radicular pain. Pain Pract.

[4] Carette S, Fehlings MG (2005). Clinical practice. Cervical radiculopathy. N Engl J Med.

[5] Ruan D, He Q, Ding Y, Hou L, Li J, Luk KD (2007). Intervertebral disc transplantation in the treatment of degenerative spine disease: a preliminary study. Lancet.

[6] Boden SD, McCowin PR, Davis DO, Dina TS, Mark AS, Wiesel S (1990). Abnormal magnetic-resonance scans of the cervical spine in asymptomatic subjects. A prospective investigation. J Bone Joint Surg Am.

[7] Wainner RS, Fritz JM, Irrgang JJ, Boninger ML, Delitto A, Allison S. Reliability and diagnostic accuracy of the clinical examination and patient self-report measures for cervical radiculopathy. Spine (Phila Pa 1976). 2003(28):52–62. 10.1097/00007632-200301010-00014 12544957

[8] Landman BA, Bogovic JA, Wan H, El Zahraa ESF, Bazin PL, Prince JL (2012). Resolution of crossing fibers with constrained compressed sensing using diffusion tensor MRI. Neuroimage.

[9] Chuanting L, Qingzheng W, Wenfeng X, Yiyi H, Bin Z (2014). 3.0T MRI tractography of lumbar nerve roots in disc herniation. Acta Radiol.

[10] Murtz P, Kaschner M, Lakghomi A, Gieseke J, Willinek WA, Schild HH, Thomas D (2015). Diffusion-weighted MR neurography of the brachial and lumbosacral plexus: 3.0 T versus 1.5 T imaging. Eur J Radiol.

[11] Gutmann L (2003). Pearls and pitfalls in the use of electromyography and nerve conduction studies. Semin Neurol.

[12] Fitzgerald RT, Bartynski WS, Collins HR (2013). Vertebral artery position in the setting of cervical degenerative disease: implications for selective cervical transforaminal epidural injections. Interv Neuroradiol.

[13] Crombe A, Alberti N, Hiba B, Uettwiller M, Dousset V, Tourdias T (2016). Cervical Spinal Cord DTI Is Improved by Reduced FOV with Specific Balance between the Number of Diffusion Gradient Directions and Averages. AJNR Am J Neuroradiol.

[14] Lawrence KE, Nabulsi L, Santhalingam V, Abaryan Z, Villalon-Reina JE, Nir TM, Ba Gari I, Zhu AH, Haddad E, Muir AM (2021). Age and sex effects on advanced white matter microstructure measures in 15,628 older adults: A UK biobank study. Brain Imaging Behav.

[15] Turna O, Turna IF (2021). Quantitative assessment of cervical spinal cord by diffusion tensor tractography in 3.0 T. Radiol Med.

[16] Martin Noguerol T, Barousse R, Socolovsky M, Luna A (2017). Quantitative magnetic resonance (MR) neurography for evaluation of peripheral nerves and plexus injuries. Quant Imaging Med Surg.

[17] Basser PJ, Pierpaoli C (1996). Microstructural and physiological features of tissues elucidated by quantitative-diffusion-tensor MRI. J Magn Reson B.

[18] Bernabeu A, Lopez-Celada S, Alfaro A, Mas JJ, Sanchez-Gonzalez J (2016). Is diffusion tensor imaging useful in the assessment of the sciatic nerve and its pathologies? Our clinical experience. Br J Radiol.

[19] Mori S, van Zijl PC (2002). Fiber tracking: principles and strategies - a technical review. NMR Biomed.

[20] Chen YY, Lin XF, Zhang F, Zhang X, Hu HJ, Wang DY, Lu LJ, Shen J (2014). Diffusion tensor imaging of symptomatic nerve roots in patients with cervical disc herniation. Acad Radiol.

[21] Lambregts DM, Beets GL, Maas M, Curvo-Semedo L, Kessels AG, Thywissen T, Beets-Tan RG (2011). Tumour ADC measurements in rectal cancer: effect of ROI methods on ADC values and interobserver variability. Eur Radiol.

[22] Ma X, Han X, Jiang W, Wang J, Zhang Z, Li G, Zhang J, Cheng X, Chen H, Guo H, Tian W (2018). A Follow-up Study of Postoperative DCM Patients Using Diffusion MRI with DTI and NODDI. Spine (Phila Pa 1976).

[23] Han X, Suo S, Sun Y, Zu J, Qu J, Zhou Y, Chen Z, Xu J (2017). Apparent diffusion coefficient measurement in glioma: Influence of region-of-interest determination methods on apparent diffusion coefficient values, interobserver variability, time efficiency, and diagnostic ability. J Magn Reson Imaging.

[24] Nogueira L, Brandao S, Matos E, Nunes RG, Ferreira HA, Loureiro J, Ramos I (2015). Region of interest demarcation for quantification of the apparent diffusion coefficient in breast lesions and its interobserver variability. Diagn Interv Radiol.

[25] Zhu F, Liu Y, Zeng L, Wang Y, Kong X, Yao S, Chen K, Jing X, Yang L, Guo X (2021). Evaluating the Severity and Prognosis of Acute Traumatic Cervical Spinal Cord Injury: A Novel Classification Using Diffusion Tensor Imaging and Diffusion Tensor Tractography. Spine (Phila Pa 1976).

[26] Hakulinen U, Brander A, Ilvesmaki T, Helminen M, Ohman J, Luoto TM, Eskola H (2021). Reliability of the freehand region-of-interest method in quantitative cerebral diffusion tensor imaging. BMC Med Imaging.

[27] Inoue C, Fujii S, Kaneda S, Fukunaga T, Kaminou T, Kigawa J, Harada T, Ogawa T (2014). Apparent diffusion coefficient (ADC) measurement in endometrial carcinoma: effect of region of interest methods on ADC values. J Magn Reson Imaging.

[28] Wu W, Yang Z, Zhang T, Ru N, Zhang F, Wu B, Liang J (1976). Microstructural Changes in Compressed Cervical Spinal Cord Are Consistent With Clinical Symptoms and Symptom Duration. Spine (Phila Pa).

[29] Wang K, Chen Z, Zhang F, Song Q, Hou C, Tang Y, Wang J, Chen S, Bian Y, Hao Q, Shen H (2017). Evaluation of DTI Parameter Ratios and Diffusion Tensor Tractography Grading in the Diagnosis and Prognosis Prediction of Cervical Spondylotic Myelopathy. Spine (Phila Pa 1976).

[30] Liang KN, Feng PY, Feng XR, Cheng H (2019). Diffusion Tensor Imaging and Fiber Tractography Reveal Significant Microstructural Changes of Cervical Nerve Roots in Patients with Cervical Spondylotic Radiculopathy. World Neurosurg.

[31] Ma C, Guo X, Liu L, Zhan Q, Li J, Zhu C, Wang L, Zhang J, Fang X, Qu J (2017). Effect of region of interest size on ADC measurements in pancreatic adenocarcinoma. Cancer Imaging.

[32] Jafari-Khouzani K, Paynabar K, Hajighasemi F, Rosen B (2019). Effect of Region of Interest Size on the Repeatability of Quantitative Brain Imaging Biomarkers. IEEE Trans Biomed Eng.

[33] Sun Y, Xiao Q, Hu F, Fu C, Jia H, Yan X, Xin C, Cai S, Peng W, Wang X (2018). Diffusion kurtosis imaging in the characterisation of rectal cancer: utilizing the most repeatable region-of-interest strategy for diffusion parameters on a 3T scanner. Eur Radiol.

